# The Influence of the Grinding Media Diameter on Grinding Efficiency in a Vibratory Ball Mill

**DOI:** 10.3390/ma17122924

**Published:** 2024-06-14

**Authors:** Paweł Tomach

**Affiliations:** AGH University of Krakow, Faculty of Mechanical Engineering and Robotics, Department of Machinery Engineering and Transport, 30-059 Kraków, Poland; tomach@agh.edu.pl; Tel.: +48-12-617-30-73

**Keywords:** raw materials, grinding process, vibratory mill, grinding media, very fine grinding, micro powders

## Abstract

The grinding process plays a crucial role in industry, allowing for the reduction of particle sizes of raw materials and substances to the required fineness—either as a finished product or for further technological processes. The high demand for micro- and nanopowders or suspensions is associated with the high energy consumption of the milling process. Therefore, optimizing the milling process, including correctly selecting grinding media, is essential to reduce energy consumption. This article presents experimental studies of the grinding process of a model material (quartz sand) in a laboratory vibratory mill. Five sets of grinding media with different diameters were used in the research, and grinding was conducted for various durations. The studies showed that the vibratory grinding process is efficient for each set of grinding media and grinding durations. The research has shown that conducting studies on the proper selection of mills is beneficial, especially regarding very fine grinding of various materials. The study confirmed that properly selecting grinding media sets can significantly accelerate the grinding process. For the selected technological variant, it was demonstrated that using 15 mm grinding media, compared to 12 mm, resulted in a 22.5% reduction in grinding time to achieve a specified particle size class of 0–10 μm.

## 1. Introduction

The vast majority of mineral raw materials, artificially produced materials, and industrial and post-consumer waste occurs in the form of granulated material. In almost every case, using each of the materials mentioned above requires grinding it to the required grain size, often below 1 mm or even up to several dozen nanometers. This grinding is carried out in various types of mills: ball mills [1], stirred mills [2,3], cylindrical mills [4,5], vibratory mills [6,7,8] and others [9,10,11,12]. The grinding energy requirement increases as the grinding product’s grain size decreases. It is estimated that comminution processes consume 4% of global energy. When considering the energy consumption of mining plants (e.g., copper ore mines and other metal ore mines), comminution processes (mainly grinding) consume approximately 50% of the plant’s energy demand [13,14].

Grinding process optimization is crucial for achieving high efficiency and performance in industrial mills. Many scientific articles focus on this topic, analyzing various aspects and utilizing different models and tools (software) to support this process [15,16,17,18]. However, these publications mainly concern ball mills [19,20], roller mills [21,22], autogenous and semi-autogenous (AG/SAG) mills [23], or stirred mills [24,25,26].

Grinding process optimization aims to improve the mill’s technological capabilities (enhancing grain size parameters, increasing throughput) and reduce the grinding process’s energy consumption, translating into lower production costs and mitigating the negative environmental impact.

In the case of mills with free grinding media (balls), one of the parameters strongly affecting the grain size parameters of the grinding product (the ability to achieve the desired particle size distribution) is the proper selection of the mill set (diameter, material from which they are made). In the literature, one can find many studies regarding the influence of the type of grinding media used on the grain size parameters of the grinding product—however, these studies mainly concern stirred mills [27,28,29,30] and ball mills [1,31,32,33,34,35,36]. It is challenging, however, to find studies regarding the influence of the type of mills used on the grinding efficiency in vibratory mills—which is the subject of this article.

This article is about grinding in a vibratory mill. Such mills are classified as mills with free grinding wheels in which energy is transferred to the grinding process through a movable (vibrating) chamber. Grinding materials are a crucial element in production processes in almost all industries using vibratory mills. Vibratory mills are utilized across various industries, from cement production to pharmaceuticals, where these machines are used to manufacture materials for a specified grain size. In most industrial vibratory mills’ designs, their chambers are set into vibrating motion using inertial vibrators. However, vibrations are forced using a kinematic vibrator (eccentric shaft, e.g., in laboratory mills). Vibration mills are machines with wide technological capabilities and very diverse structures. They are used as laboratory mills with a power of up to several kW and, mostly, as industrial mills with a power of several dozen kW to 2000 kW [37,38].

The grinding process in vibratory and ball mills occurs between the grinding media, usually balls, and between the grinding media and the chamber lining. Grinders with other shapes, such as cylinders or rods, are rarely used. In tubular vibratory mills, the chamber has the shape of a cylinder, closed on both ends with sieve partitions, retaining the grinding media and allowing the ground material to pass through with air (in dry grinding) or water (in wet grinding). In some mills, the feed is introduced directly into the chamber. Then, the chamber has one sieve partition located on the side of the outlet of the ground material, and in the case of rod grinding, the chamber has no sieve partitions. In the vast majority of cases, especially fine grinding [39,40,41], the set of grinding media is usually composed of balls of the exact dimensions. However, the grinding media wear out during the grinding, causing their dimensions to vary.

The dominant majority of vibratory mills are tubular mills whose chambers perform vibrating motion with a vibration amplitude trajectory close to circular or elliptical. In most designs, they are vibrating machines—super-resonant, with vibrations forced using inertial vibrators—just like the widely known and used vibrating screens [42]. The efficiency of industrial mills, determined by their use, the type of ground material, grinding conditions, and grain size of the feed and grinding product, ranges from several dozen kg/h to 60 Mg/h [38]. Several companies manufacture vibration mills in the USA, Germany, France, Russia, Japan, China, the Czech Republic, Turkey, Kazakhstan and Korea.

A significant difference between the operation of a ball mill and that of a vibratory mill is the method of transmitting energy to the grinding media. In a ball mill, the grinding media receive energy from the rotating chamber, and their free movement is only caused by the force of gravity. In vibratory mills, grinding media obtain energy from the vibrating chamber, and it can be increased by increasing the frequency and amplitude of vibrations of the mill working unit in which one or more chambers are installed.

## 2. Comminution in Vibratory Mills with Free-Flowing Grinding Media—Elements of Theory

**Figure 1:**
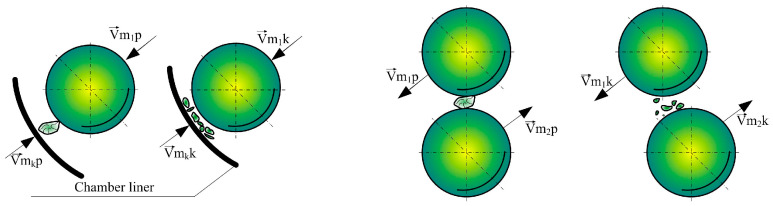
Scheme of material grinding between grinding media and the chamber and between two grinding media. v_m1p_, v_m2p_—initial speeds of grinding media; v_m1k_, v_m2k_—final speeds of grinding media; v_pk_, v_kk_—initial, final speed of the mill chamber.

In vibratory mills, the grinding process occurs mainly between grinding media colliding with each other and between the grinding media and the chamber lining. The most commonly used grinding media are ball-shaped, while other shapes, such as rods, cylinders or others, are rare. The scheme of grinding the material between the balls and between the balls and the chamber is shown in Figure 1. The diagram schematically presents the variants of material grinding in this type of mill: the ground material is located between the chamber and the grinder or between two grinders. The schematic representation of the speeds of the grinders and the chamber before and after collision requires the determination of complex motion equations, which take into account various coefficients (elasticity, restitution, damping, friction, and other material parameters) as well as the most challenging, the dissipation of energy during the grinding process. Equations of this type, describing the behavior of the grinders in the mill chamber or computer simulations, have been the subject of other research studies [1,43,44,45]. A set of grinding media typically comprises balls of uniform dimensions. However, it is common that during operation, there is an automatic, uneven differentiation of their dimensions, resulting in a mixture of balls with varying sizes.

**Figure 2:**
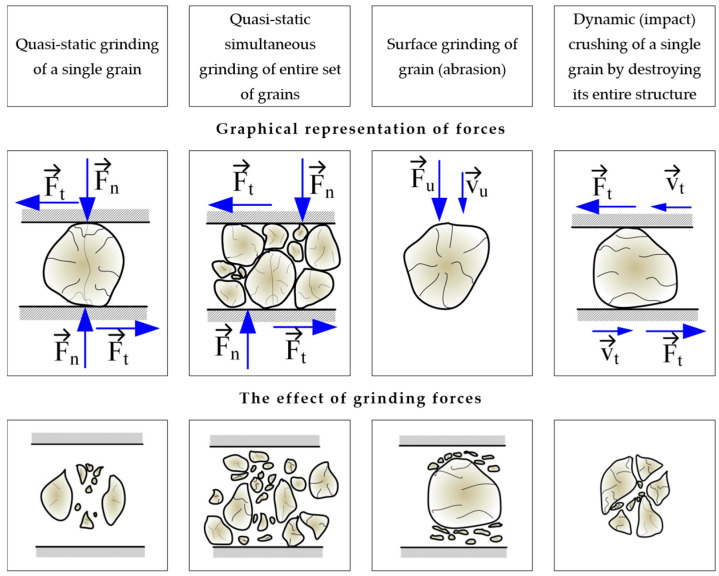
General comminution cases occurring in vibratory mills. F_t_—tangential force (friction), F_n_—normal force, F_u_—impact force, v_t_—direction of friction velocity, v_u_—direction of impact velocity.

In vibratory mills, grinding non-plastic (brittle) materials occurs on a microscopic and submicroscopic scale. The development of technologies enabling nano-materials [46] and the processes used in disposal and recycling make grinding on a sub-microscopic scale increasingly important. Considering the process of grinding brittle materials in vibratory mills, from the perspective of the machine and its working grinding elements and the dynamics of material cohesion disintegration, four general cases of grinding can be distinguished, as presented in Figure 2.

Various disintegration models were used to describe the process of grinding material grains between grinding media [45,47]. We can distinguish here, for example, the models by Schönert [48] and two models by Yokoyama et al. [46].

**Figure 3:**
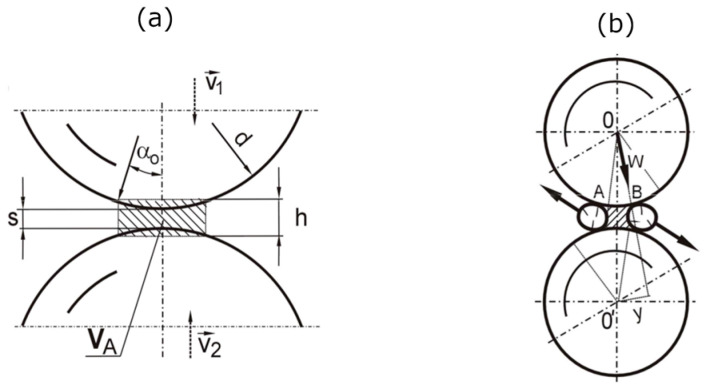
The elementary mechanisms of mechanical comminution occurring in mills with grinding balls: (**a**) the general case given by Joisel; (**b**) grinding mechanism according to Schönert [48]. d—diameter of the balls; v_1_, v_2_—impact velocities of the grinding media; V_A_—volume of the grain collection between the grinding media; h—height of the grain collection; s—distance between the grinding media during the collision; α_0_—angle between the axis of both grinding wheels and the radius.

Joisel assumed the collisions of balls of the same diameter as the central collisions, and the material distribution during grinding was as shown in Figure 3b. Schönert, however, assumed that between the balls there is a collection of grains from which, when the balls approach each other, no grains will escape (Figure 3a). The volume V_A_ of the set of grains that are crushed according to Schönert is calculated from the expression:

(1) $$V_{A}=\frac{\pi }{4}h{\left(\alpha_{0}d\right)}^{2}$$

where:

V_A_: volume of grain collection between grinders, m^3^,

h: grain harvest height, m,

d: ball (grinding) diameter, m,

α_0_: angle between the axis of both grinding media and the radius, rad.

The angle α_0_ has values in the range of 0.08 ÷ 0.12 rad and depends on the friction coefficient between the material being ground and the material of the grinding media and, for the most frequently ground materials, is in the range of 0.25 ÷ 0.35. For such values of the friction coefficient, the ratio of the diameter of the grinding media (balls) to the maximum grain size oscillates in the range of 16 ÷ 32.

Schönert showed that for grinding brittle materials with grain shapes close to a ball, the intensity of the material grinding process between the grinding media is variable and depends on the volume of the grains between the grinding media and their speed. According to Schönert, the value of energy spent on the grinding process can be expressed as:

(2) $$E_{M}=\frac{E_{V_{1,2}}\left(\frac{V_{km}}{V_{p}}\right)}{\left.\rho_{n}\left(1-e_{e}\right)\right.}$$

where:

E_M_: energy absorbed for the grinding process, J,

E_V1,2_: energy of grinding media, J,

V_km_: volume of the grinding medium: ball, m^3^,

V_p_: volume of empty spaces between grains, m^3^,

ρ_n_: density of the ground material, kg/m^3^,

e_e_: material porosity, -.

**Figure 4:**
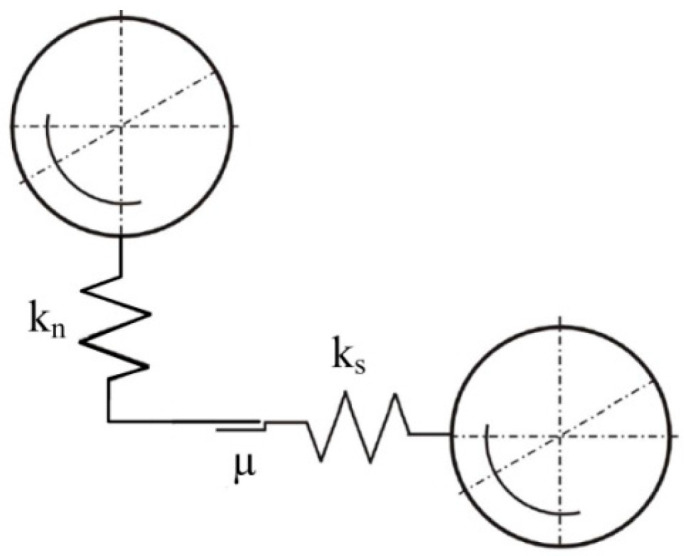
Model of ball collision in a vibratory mill according to Inoue and Okaya [48]: k_n_, k_s_—elasticity coefficient of the material in normal and tangential impact; µ—sliding friction coefficient.

The literature also includes models of ball collisions that occur in vibratory mills. These include the models by Inoue and Okaya (Figure 4) [49], Yokoyama, Tamura, Usi and Jimbo [45], as well as Michalczyk and Cieplok [43]. Inoue and Okaya developed a model and simulated it based on the discrete element method (DEM), which can be used to determine the motion of balls (grinding media) and estimate the dissipation energy—based on ball-to-ball collisions (normal forces), ball–ball (tangential–frictional forces) and ball–chamber (normal and tangential forces). This model (Figure 4) concerns the family of planetary and vibratory mills and considers the movement of balls in two directions: vertical and horizontal (tangential).

In the presented model, the grinding resistance between the balls is written as:

(3) $$R_{max}=µF$$

where:

R_max_: maximum resistance to crushing the material between the balls, N/m^2^,

µ: sliding friction coefficient, -,

F: unit pressure between the balls and the balls and the chamber, Pa.

**Figure 5:**
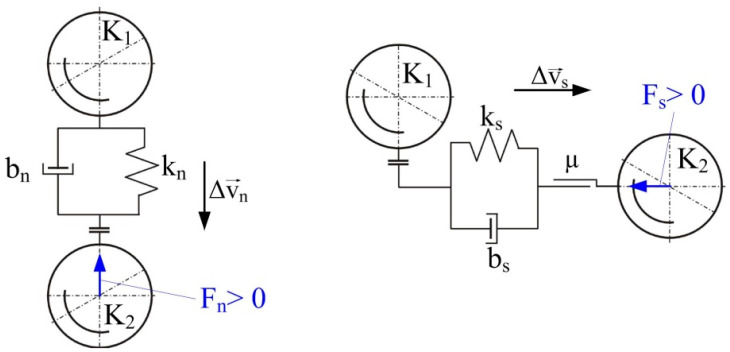
The collision model of grinding media according to Yokoyama et al. [45]. K_1_, K_2_—grinding media; k_n_, k_s_—elasticity coefficient in normal and tangential impact; b_n_, b_s_—viscous damping coefficient in normal and tangential impact; µ—sliding friction coefficient; F_n_, F_s_—normal and tangential force.

Based on the developed model, Inoue and Okaya conducted a simulation in which energy demand was determined. They verified the simulation experimentally by grinding coal, quartz and limestone [49]. A more accurate and complex model, considering two variants of collisions (central—with elasticity and contact damping, and tangential—taking into account sliding friction), was developed by Yokoyama, Tamura, Usoi and Jimbo [45]. A graphical representation of the collision, as mentioned in earlier variants, is shown in Figure 5.

Based on this model, Yokoyama, Tamura et al. [45] presented equations describing the forces acting on the i-th ball in the direction of the x and y axes. These equations contain the components of the forces coming from the remaining balls, the gravitational force and the drag and buoyancy forces—coming from the fluid surrounding the balls. They expressed the resistance force in the following form:

(4) $$F_{D}=b_{we}C_{D}\left(\frac{\pi }{4}d^{2}\right)\left(\frac{\rho_{L}}{2}v_{R}^{2}\right)\;$$

where:

F_D_: total drag force, N, 

C_D_: drag coefficient depends on the Reynolds number (Re), 

C_D_ = (0.55 + 4.8(Re)^−0.5^)^2^, -,

b_we_: correction factor taking into account the resistance on the side walls of the chamber and the liquid flow—determined experimentally, -,

d: grinding media (ball) diameter, m, 

ρ_L_: density of the fluid surrounding the balls, kg/m^3^.

As can be seen from the various models presented above, the diameter of the grinding media and the type of material from which they are made are the most important parameters influencing the energy of the grinding media. The energy of the grinding media plays a crucial role in the comminution process and directly translates into the disintegration of grains (grinding). The grinding media size will determine how many of them will be in the chamber (while maintaining the same degree of chamber filling), what amount of material will fit between them, and, therefore, indirectly—what type of grinding forces will dominate in the grinding process. The influence of the size or type of grinding media used on the grinding process parameters is a fundamental issue—and it is the subject of work by other researchers [19,50]. 

The development of new simulation programs and the increase in computational power of computers allow for more precise determination of the parameters of mill motion and their energy. Developing a mathematical description that considers all factors influencing the energy of mills and the comminution process is a complex and demanding task. The use of CAD computer programs, based on DEM and CFD methods, becomes significant when attempting to model comminution processes in mills. Proper selection of numerous mechanical parameters, such as compressive strength, Young’s and Poisson’s moduli, as well as various contact parameters like friction coefficients, damping, restitution for different types of contacts between mills, chamber, and milled material, and appropriate modelling of the milling environment (especially in the case of milling in liquids), allows for obtaining reliable energy data of the milling process after validating the adopted parameters [51,52,53,54]. Among other things, simulation studies aiming to determine the energy of mills, their trajectories, or energy requirements for the comminution process in ball mills and stirred mills are the subject of numerous scientific works [3,15,26,55,56,57,58]. These methods can successfully be implemented to model the comminution process in vibratory mills. Due to the significant energy consumption of very fine grinding processes, which can also be successfully carried out in vibratory mills, it is necessary to look for solutions to reduce the energy consumption of these processes—which certainly also includes the proper selection of a set of grinding media.

## 3. Description of Test Stands—Materials and Methods

### 3.1. Test Stand for a Vibratory Mill

**Figure 6:**
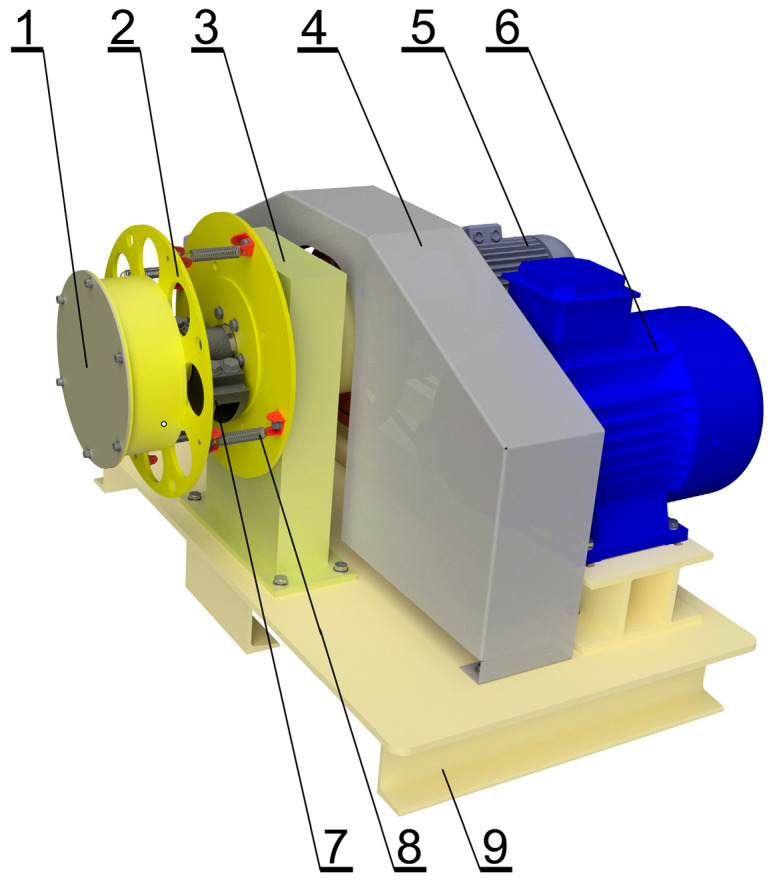
View of the test stand of a vibratory mill with periodic operation. 1—chamber with a capacity of 2.2 dm^3^; 2—disk (chamber mounting); 3—supporting structure and bearing of the vibrator shaft; 4—belt drive cover; 5—drive motor for chamber rotation (not used in the experiments); 6—drive motor for the vibrator shaft; 7—eccentric shaft (vibrator) with balancing mass; 8—elastic elements preventing chamber rotation around its own axis; 9—supporting frame.

Experimental tests of the grinding process were carried out in a laboratory vibratory mill with periodic operation, a view of which is shown in Figure 6.

Forcing the chamber to vibrate in this mill (Figure 6) is achieved through a kinematic vibrator (7) (causing a circular trajectory of chamber vibrations), which is an eccentric shaft with an adjustable eccentric radius. At the end of the shaft, there is a disc (2) mounted (with bearings) to which the mill chamber (1) is attached using screw connections. The eccentric shaft (7) is rotated through a belt transmission transmitting the rotational speed from the vibrator drive motor (6). The rotational speed of the output shaft of the vibrator motor (and therefore, indirectly, the rotational speed of the vibrator shaft) is regulated by a frequency converter included in the control system (not visible in Figure 6). 

The basic operating parameters of the vibratory mill during the research are presented in Table 1.

### 3.2. The Course of Grinding Tests

The laboratory vibratory mill station was set up to conduct grinding process tests in accordance with the established research plan. Utilizing the adjustable eccentricity radius of the vibrator shaft, the amplitude of chamber vibrations was configured. Additionally, an inverter powering the vibrator drive motor enabled precise control over the vibration frequency of the working unit. A representative sample of quartz sand was extracted using a sample divider for subsequent laboratory grinding tests. The mass of the load—comprising sand and grinding media poured into the mill chamber—was determined using the following formula:

(5) $$m_{ł}=m_{m}+m_{k}$$

(6) $$m_{m}=k_{k}\cdot b\cdot V_{k}\cdot \rho_{m}$$

(7) $$m_{k}=b\cdot V_{k}\cdot \rho_{k}$$

where:

m_ł_: load mass, kg,

m_m_: material weight, kg,

m_k_: mass of grinding media (balls), kg,

k_k_: grinding media arrangement factor, k = 0.4, -,

*b*: chamber filling level, -,

*V_k_*: chamber volume, dm^3^

*ρ_k_*: bulk density of grinding media, kg/dm^3^,

*ρ_m_*: bulk density of the material, kg/dm^3^.

The prepared amount of material and grinding media was introduced into the chamber of the laboratory vibratory mill. Then, after closing the chamber, it was attached to the mill disc. Appropriate times were set to perform grinding kinetics. After grinding was completed, the chamber was dismantled, and a sample of ground material was taken from various places in the chamber. After these operations, the chamber was emptied and cleaned and then filled with the charge again, continuing the research on the grinding process for longer grinding times. Similarly, samples were taken to test the grain size of the grinding product during these times.

The variable parameters during the research on the quartz sand grinding process were the grinding time and the set of grinding media. Five sets of grinding media were used for the tests—identical steel balls with 10.0, 12.0, 13.5, 15.0 and 17.5 mm diameters. In order to determine the grinding kinetics, the following times were assumed: 2.5, 5.0, 10.0 and 20.0 min. Additionally, for grinders with diameters of 10 and 17.5 mm, grinding lasting 40 min was performed.

### 3.3. Grain Size Distribution Analysis

**Figure 7:**
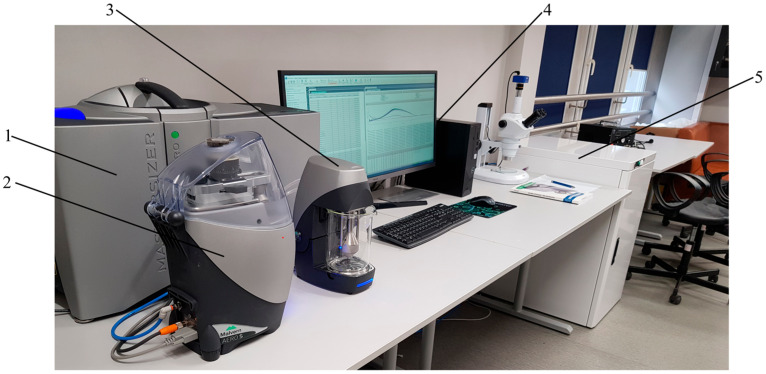
Research stand for grain size measurement—Malvern Mastersizer 3000. 1—main measuring unit; 2—dry dispersion unit (Aero S); 3—wet dispersion unit (Hydro EV); 4—measurement computer with monitor; 5—compressed air system (for dry measurements).

The grain size distribution analysis of feedstock and milling products followed the ISO 13320:2020 standard [59]. The analysis was conducted using a laser grain size analyzer, Malvern Mastersizer 3000 (Malvern Panalytical LTD, Malvern, United Kindgom). The measurements were performed via dry measurement method in an air environment, using the dispersing unit ‘Aero S’. The utilized surface dispersion module (Aero S) enables the characterization of granular materials over a wide measurement range from 0.01 to 3500 µm. Malvern Mastersizer version 3.81 software was used to measure and analyze data. In the analysis of results, the device employs two scattering models: the Fraunhofer approximation and the Mie theory. The research apparatus setup is shown in Figure 7, and the measurement parameters used are presented in Table 2.

The study examined the grain size parameters, including dv(10), dv(50), dv(90), and the percentage of grain classes below 100, 50 and 10 µm. The measurements were carried out five times for each sample, and the results presented in the article are the average of these measurements.

### 3.4. Materials Utilized in the Study

The material selected for the study was quartz sand, glass grade I, sourced from the Sand Mine in Osiecznica, Poland. This material was chosen as a model material due to its high grain stability, purity, chemical resistance, non-hygroscopic properties, and its common use in research on milling processes in vibratory mills and other mills with diverse designs. Below are the basic parameters of the measured quartz sand used for the study:

− specific density: 2.66 kg/dm^3^,
− bulk density: 1.50 kg/dm^3^,
− grain size dv(10): 242 μm,
− grain size dv(50): 398 μm,
− grain size dv(90): 643 μm.

**Figure 8:**
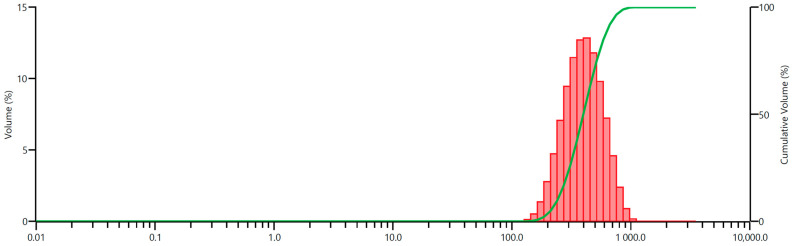
Grain size parameters of the feed (quartz sand before milling): histogram and undersize.

The cumulative undersize curve and grain size distribution histogram of the feed are presented in Figure 8.

## 4. Experimental Research on the Grinding Process—Results and Discussion

Below, in Table 3, symbols representing samples taken for grain size analysis for individual sets of mills and milling times are presented. In a subsequent part of the article, these samples will be described using these symbols with the prefix ‘average’ in the legends of the graphs, as the results presented in the graphs are the average of the measurements conducted.

**Figure 9:**
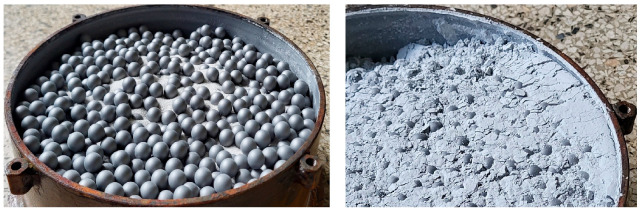
View of the chamber with 17.5 mm grinding media and material before grinding (**on the left**) and after 40 min of grinding (**on the right**).

A view of the chamber with 17.5 mm grinding media and the material before grinding is presented on the left side of Figure 9, while the result of 40 min of grinding is shown on the right side. The image showing the ground product in the chamber depicts an increase in material volume during grinding, resulting from high comminution efficiency (lower bulk density of this finer-grained material). In the case of very fine grinding, it is periodically necessary to consider the change in bulk density of the material during grinding to avoid excessive chamber filling with material, as this leads to a decrease in grinding speed.

**Figure 10:**
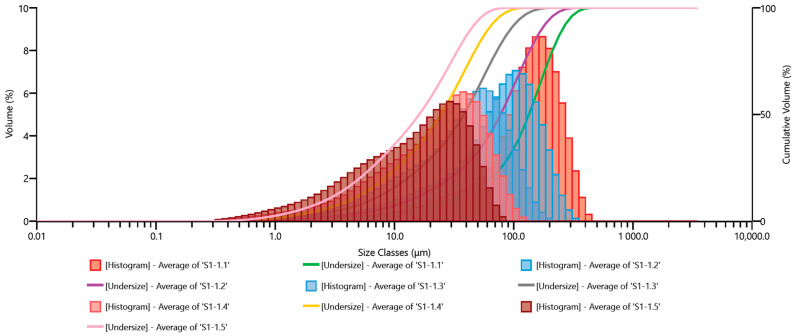
Cumulative size distribution curve of the milling product and grain size distribution histogram—set of grinding media with a diameter of 10.0 mm.

The grain size analysis results are presented as grain size distribution histograms (percentage share) and cumulative curves for the set of Ø10.0 mm grinding media and the adopted milling times in Figure 10.

**Figure 11:**
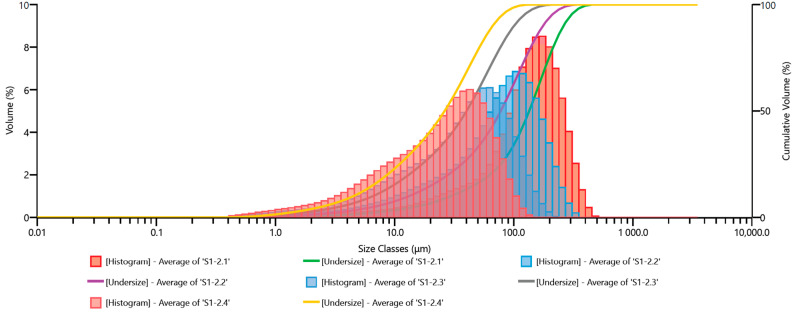
Cumulative size distribution curve of the milling product and grain size distribution histogram—set of grinding media with a diameter of 12.0 mm.

**Figure 12:**
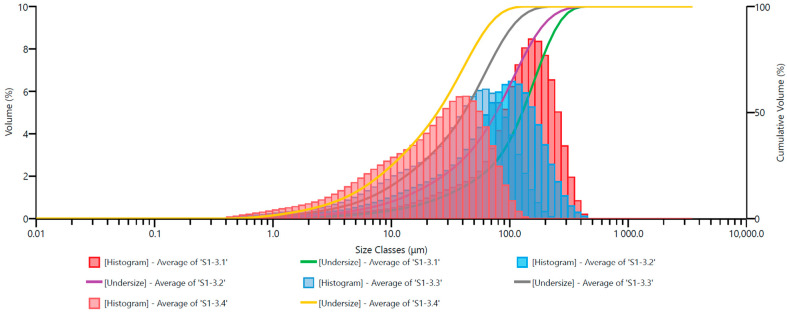
Cumulative size distribution curve of the milling product and grain size distribution histogram—set of grinding media with a diameter of 13.5 mm.

**Figure 13:**
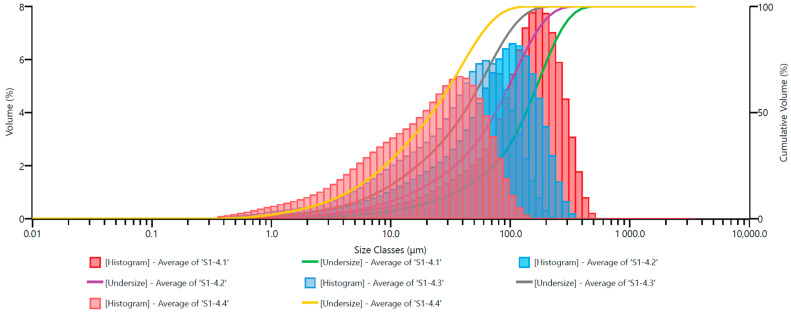
Cumulative size distribution curve of the milling product and grain size distribution histogram—set of grinding media with a diameter of 15.0 mm.

**Figure 14:**
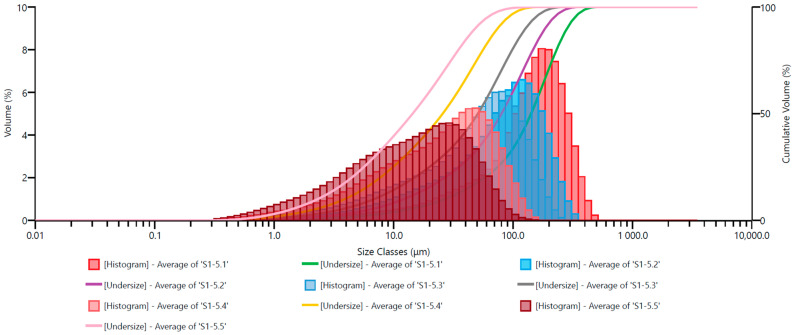
Cumulative size distribution curve of the milling product and grain size distribution histogram—set of grinding media with a diameter of 17.5 mm.

In the subsequent figures, similar grain size distribution plots are depicted: Figure 11 for Ø12.0 mm grinding media; Figure 12 for Ø13.5 mm grinding media; Figure 13 for Ø15.0 mm grinding media; and Figure 14 for Ø17.5 mm grinding media. To facilitate the interpretation of the graphs, the grain size distribution of the quartz sand before milling (feedstock) was not included.

The analysis of the grain size distribution curves for different grinding media diameters provides valuable insights into the effect of varying grinding media sizes on the resulting particle size distribution. Comparing the plots allows for a comprehensive understanding of how changes in grinding media diameter influence the milling process. Additionally, observing the distribution trends across different media sizes aids in optimizing grinding parameters for desired particle size outcomes.

By excluding the initial grain size distribution of the feedstock, the focus remains solely on the impact of the milling process itself. This approach enables a more precise assessment of the effectiveness of the milling operation in achieving the desired particle size reduction. Furthermore, it allows for a more direct evaluation of the efficiency of the chosen grinding media diameter in achieving the desired particle size distribution.

The presented graphs indicate that the milling process of quartz sand in the vibratory mill is highly efficient for each of the adopted sets of grinding media. Proper interpretation of the graphs mentioned above (Figure 10, Figure 11, Figure 12, Figure 13 and Figure 14) allows us to conclude that the grinding media influences the grain size distribution of the milling product. These differences, especially for shorter milling times, are relatively small—the cumulative curves for different sets of grinding media are very similar, and detecting differences in grain size may be challenging. Therefore, comparing them for the exact milling times may give the erroneous impression that the changes in grain size for different technological variants (used grinding media) are minor. Hence, especially for fine milling, it is crucial to analyze the percentage shares of fine grain classes in the milling product—presented, among others, by histograms—and in this case, it is evident that depending on the set of grinding media used, the shares of these grain classes vary.

**Figure 15:**
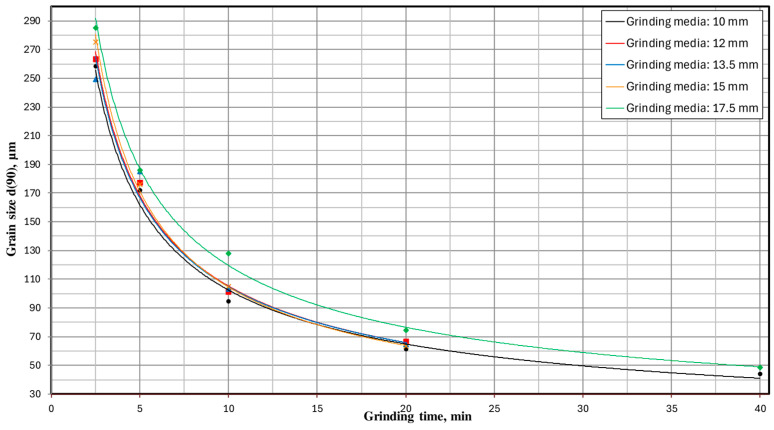
The influence of milling time and the type of grinding media used on the particle size d(90).

**Figure 16:**
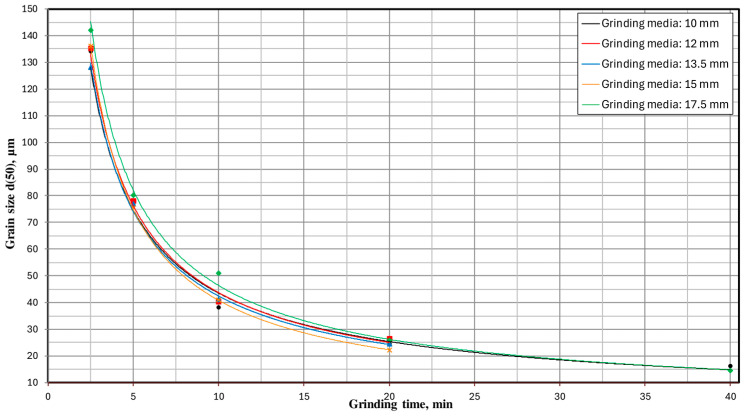
The influence of milling time and the type of grinding media used on the particle size d(50).

**Figure 17:**
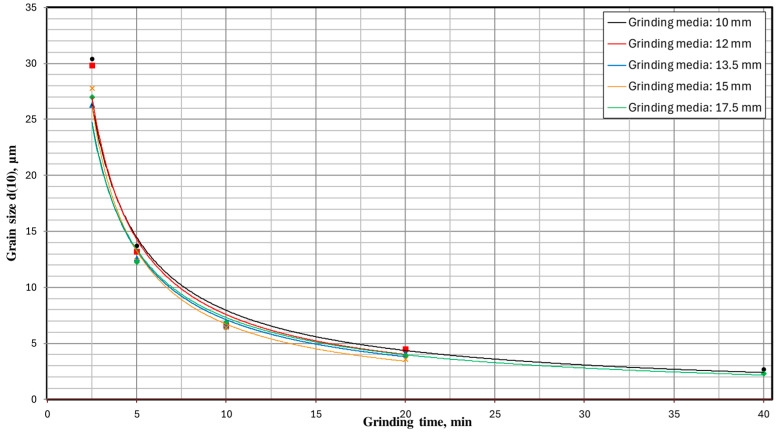
The influence of milling time and the type of grinding media used on the particle size d(10).

In order to fully visualize the results of the conducted research and increase their clarity, the obtained results are presented in the form of milling kinetics. Figure 15 shows the milling kinetics regarding the influence of milling time and the set of grinding media on the particle size d(90). These relationships for grain sizes d(50) and d(10) are presented accordingly in Figure 16 and Figure 17.

**Figure 18:**
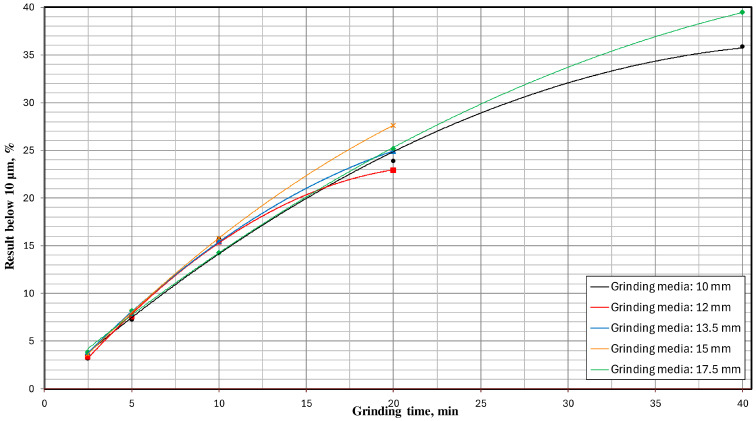
The influence of milling time and types of grinding media on the percentage of particles on the 0–10 μm grain size class.

Below, Figure 18 depicts a different type of milling kinetics—the influence of the type of grinding media and milling time on the percentage share of the grain size class below 10 µm—while maintaining the same mill operating parameters (vibration frequency and amplitude) and technological parameters (chamber filling degree and milling time). 

The milling kinetics are significantly influenced by the physical properties of the material. The nanomechanical properties become particularly important when grinding the material to the nanoscale [60]. Additionally, the initial grain size of the feed material is a critical factor that affects the efficiency and outcome of the milling process [61]. The presented milling kinetics (Figure 18) demonstrate a significant influence of the type of grinding media on the grain size distribution of the milling product—the greater the fineness of the desired grain size, the more significant the impact. This is evidenced by, for example, the percentage share of the class below 10 μm obtained for a milling time of 20 min, where using 15 mm grinding media resulted in a 16% higher share of this grain size class than with 12 mm grinding media. Looking at it another way, hypothetically, if there were a technological target to achieve a percentage share of this grain size class of 23%, then with 12 mm grinding media, the milling process would need to last 20 min. At the same time, with 15 mm grinding media, it would be around 15.5 min—i.e., 22.5% less, directly translating to 22.5% less energy consumption to produce such a milling product. Significant differences can also be observed when comparing the results obtained for a milling time of 40 min. In this case, using 10 mm grinding media enabled the attainment of the analyzed grain fraction at 35.9%, whereas, for 17.5 mm grinding media, this value was 39.5%—representing a result 10% more favorable. Similarly, hypothetically, if the technological goal of the production plant were to achieve a grain size class of 0–10 μm in a quantity of 36%, then using the appropriate size of grinding media (in this case, 17.5 mm) would allow us to achieve this goal after only 33.5 min of milling—i.e., 19% faster than if 10 mm grinding media were used.

The complete set of grain size distribution parameters for the milling products for each set of grinding media and milling time, such as grain size d(10), d(50), d(90), and percentage shares of grain classes 0–100 μm, 0–50 μm and 0–10 μm, are presented in Table 4.

The results of grain size analysis presented in the above table allow for the assessment of milling efficiency according to various technological indicators. One of such indicators is the degree of comminution, S_x_, defined by the expression:

(8) $$S_{x}=\frac{F(x)}{P(x)}$$

where:

S_x_: x-th degree of fragmentation, -,

F(x): grain size d(x) of the feed, µm,

P(x): grain size d(x) of the grinding product, µm,

For example, for a milling time of 20 min, the degrees of comminution S_10_, S_50_ and S_90_ were:

− for Ø 10.0 mm grinding media: S_10_ = 53; S_50_ = 16; S_90_ = 10;
− for Ø 12.0 mm grinding media: S_10_ = 54; S_50_ = 15; S_90_ = 10;
− for Ø 13.5 mm grinding media: S_10_ = 59; S_50_ = 16; S_90_ = 10;
− for Ø 15.0 mm grinding media: S_10_ = 67; S_50_ = 18; S_90_ = 10;
− for Ø 17.5 mm grinding media: S_10_ = 62; S_50_ = 15; S_90_ = 9.

The above-presented grain size results allow for a broad interpretation. For those interested in fine milling, this data enables the determination of the impact of grinding media type on milling process parameters, depending on preferred grain size classes and the significance of comminution degrees for a given application. Therefore, the article did not present other technological indicators for various milling times to avoid unnecessarily increasing its volume.

## 5. Conclusions

The article presents the results of fine milling experiments on quartz sand with a grain size of 100% below 1 mm using a laboratory vibratory mill with low-frequency vibrations. These studies aimed to determine whether the diameter of the applied grinding media (steel balls) affects the grain size parameters of the milling product—and thus the efficiency and energy consumption of the process. Five sets of grinding media with diameters ranging from 10.0 to 17.5 mm were used in the experiments. From the conducted research, the following conclusions can be drawn:

− The research demonstrated high efficiency in the milling process of quartz sand in the vibratory mill—as evidenced by the grain size parameters of the milling products and the achieved degrees of comminution.
− The obtained research results indicate that the type of grinding media used significantly affects the grain size of the milling product—especially in fine and very fine grinding (below 10 µm).
− Analysis of the percentage share of the grain size class below 10 μm revealed that for a milling time of 20 min, the finest grain size was obtained using grinding media with a diameter of 15 mm. This set of grinding media allowed for obtaining 16% more of this grain size class at the same time compared to grinding media with a diameter of 12 mm. As shown earlier, this translates into even 22.5% less energy consumption.
− Two milling variants for a milling time of 40 min showed that if the technological goal of the production plant was to achieve a grain size class of 0–10 μm in an amount of 36%, the use of the appropriate size of grinding media—in this case 17.5 mm—would allow achieving this goal 19% faster compared to using grinding media with a diameter of 10 mm. Moreover, this would be associated with 19% less energy consumption.
− The finer the grain size we aim to achieve in the comminution process, the greater the energy demand. In the case of very fine milling, proper selection of grinding media allows for a significant reduction in energy consumption, positively impacting the natural environment.
− The examples of comminution degrees and the provided data on grain size parameters showed that grinding media with a diameter of 15.0 mm was the most favorable set.

There are no research results on vibrating mills in the literature that cover topics similar to those presented in this article. The presented experimental research results are new and unique in this field. The conclusions emphasize the importance of the proper selection of grinding media for energy efficiency and technological possibilities.

The results presented in the article clearly indicate that the selection of the appropriate set of grinding media significantly impacts the milling process’s efficiency. Analysis of the percentage shares of fine grain size classes and the presented milling kinetics demonstrate that the proper selection of grinding media can significantly reduce energy consumption during the milling process.

The research findings indicate the potential for very fine comminution in vibratory mills and their high efficiency in grinding various materials. Vibratory mills are characterized by their high versatility, and the selection of the appropriate type of mill should be conducted on a laboratory scale before the design or selection of an industrial mill.

## Figures and Tables

**Table 1:** Basic parameters of the vibratory mill test stand.

Parameter Name	Unit	Value
Amplitude of chamber vibrations	mm	10
Chamber vibration frequency	Hz	12
The degree of filling of the chamber	%	80
Vibrator drive motor power	kW	3.5
Chamber diameter	mm	210
Chamber volume	dm^3^	2.2

**Table 2:** List of adopted grain size measurement parameters using the dry method.

Parameter Name	Unit	Value
Particle type	-	Non-spherical
Material: refractive index	-	1.543
Material: absorption index	-	0.01
Material: density	g/cm^3^	2.66
Sample dispersion: Air pressure	bar	1.0
Sample dispersion: Feed rate	%	50
Sample dispersion: Venturi type	-	Standard Venturi disperser
Sample dispersion: Try type	-	General purpose tray Hopper gap: 1 mm
Analysis model	-	General purpose

**Table 3:** Symbols utilized for the samples collected for grain size analysis.

Diameter of Grinding Media		Grinding Time			
	2.5 min	5.0 min	10.0 min	20.0 min	40.0 min
Ø10.0 mm	S1–1.1	S1–1.2	S1–1.3	S1–1.4	S1–1.5
Ø12.0 mm	S1–2.1	S1–2.2	S1–2.3	S1–2.4	
Ø13.5 mm	S1–3.1	S1–3.2	S1–3.3	S1–3.4	
Ø15.0 mm	S1–4.1	S1–4.2	S1–4.3	S1–4.4	
Ø17.5 mm	S1–5.1	S1–5.2	S1–5.3	S1–5.4	S1–5.4
Quartz sand feed		“Quartz sand—feed”			

**Table 4:** Compilation of selected grain size parameters from all grinding samples.

Grinding Media, mm	Grinding Time, min	Sample Name	Grain Size, µm			Result Below 100 μm, %	Result Below 50 μm, %	Result Below 10 μm, %
			d(10)	d(50)	d(90)			
-	0	Quartz sand—feed	242.0	398.0	643.0	0.0	0.0	0.0
Ø10.0	2.5	Average of ‘S1–1.1’	30.4	134.0	258.0	33.9	15.9	3.2
	5	Average of ‘S1–1.2’	13.7	77.7	172.0	63.2	32.4	7.3
	10	Average of ‘S1–1.3’	6.5	38.2	94.5	91.8	62.2	15.7
	20	Average of ‘S1–1.4’	4.3	24.4	61.4	99.3	82.1	23.9
	40	Average of ‘S1–1.5’	2.7	16.1	43.9	100.0	93.9	35.9
Ø12.0	2.5	Average of ‘S1–2.1’	29.8	135.0	263.0	34.0	16.1	3.4
	5	Average of ‘S1–2.2’	13.2	77.9	177.0	62.7	32.9	7.5
	10	Average of ‘S1–2.3’	6.6	40.2	101.0	89.8	59.5	15.4
	20	Average of ‘S1–2.4’	4.5	26.3	66.6	98.6	78.5	22.9
Ø13.5	2.5	Average of ‘S1–3.1’	26.3	128.0	249.0	36.9	17.9	3.9
	5	Average of ‘S1–3.2’	12.6	76.9	185.0	62.7	33.8	7.9
	10	Average of ‘S1–3.3’	6.6	41.1	103.0	89.0	58.5	15.5
	20	Average of ‘S1–3.4’	4.1	24.5	64.4	98.9	80.4	24.9
Ø15.0	2.5	Average of ‘S1–4.1’	27.8	136.0	275.0	35.2	17.5	3.6
	5	Average of ‘S1–4.2’	12.4	75.4	176.0	63.7	34.5	8.1
	10	Average of ‘S1–4.3’	6.4	40.9	105.0	88.5	58.4	15.8
	20	Average of ‘S1–4.4’	3.6	22.2	63.1	98.7	82.0	27.6
Ø17.5	2.5	Average of ‘S1–5.1’	27.0	142.0	285.0	33.7	17.6	3.9
	5	Average of ‘S1–5.2’	12.3	80.1	186.0	60.4	33.3	8.2
	10	Average of ‘S1–5.3’	6.9	50.8	128.0	80.6	49.4	14.3
	20	Average of ‘S1–5.4’	3.9	25.8	74.5	96.9	75.3	25.2
	40	Average of ‘S1–5.5’	2.3	14.4	48.4	99.6	90.9	39.5

## Data Availability

The original contributions presented in the study are included in the article, further inquiries can be directed to the corresponding author.
